# The Effects of Choline on Hepatic Lipid Metabolism, Mitochondrial Function and Antioxidative Status in Human Hepatic C3A Cells Exposed to Excessive Energy Substrates

**DOI:** 10.3390/nu6072552

**Published:** 2014-07-09

**Authors:** Jie Zhu, Yang Wu, Qingya Tang, Yan Leng, Wei Cai

**Affiliations:** 1 Department of Clinical Nutrition, Xin Hua Hospital Affiliated to Shanghai Jiao Tong University School of Medicine, Shanghai 200092, China; E-Mails: tangqingya@163.com (Q.T.); caiw1978@126.com (W.C.); 2 Key Laboratory of Pediatric Gastroenterology and Nutrition, Shanghai Institute for Pediatric Research, Xin Hua Hospital Affiliated to Shanghai Jiao Tong University School of Medicine, Shanghai 200092, China; 3 Department of Nutrition, Shanghai Jiao Tong University School of Medicine, Shanghai 200025, China; 4 Department of Anesthesiology, Renmin Hospital of Wuhan University, Wuhan 430060, China; E-Mails: wywytwin@163.com (Y.W.); lengyan890801@sina.cn (Y.L.)

**Keywords:** choline, hepatic lipid metabolism, mitochondria, proliferator-activated receptor alpha, methylation

## Abstract

Choline plays a lipotropic role in lipid metabolism as an essential nutrient. In this study, we investigated the effects of choline (5, 35 and 70 μM) on DNA methylation modifications, mRNA expression of the critical genes and their enzyme activities involved in hepatic lipid metabolism, mitochondrial membrane potential (Δψm) and glutathione peroxidase (GSH-Px) in C3A cells exposed to excessive energy substrates (lactate, 10 mM; octanoate, 2 mM and pyruvate, 1 mM; lactate, octanoate and pyruvate-supplemented medium (LOP)). Thirty five micromole or 70 μM choline alone, instead of a low dose (5 μM), reduced hepatocellular triglyceride (TG) accumulation, protected Δψm from decrement and increased GSH-Px activity in C3A cells. The increment of TG accumulation, reactive oxygen species (ROS) production and Δψm disruption were observed under LOP treatment in C3A cells after 72 h of culture, which were counteracted by concomitant treatment of choline (35 μM or 70 μM) partially via reversing the methylation status of the peroxisomal proliferator-activated receptor alpha (PPARα) gene promoter, upregulating PPARα, carnitine palmitoyl transferase-I (CPT-I) and downregulating fatty acid synthase (FAS) gene expression, as well as decreasing FAS activity and increasing CPT-I and GSH-Px activities. These findings provided a novel insight into the lipotropic role of choline as a vital methyl-donor in the intervention of chronic metabolic diseases.

## 1. Introduction

Choline is an essential nutrient for human health, which exerts various physiological functions: (1) it is acetylated to generate acetylcholine, the important neurotransmitter; (2) it is oxidized to pass methyl to *S*-adenosylmethionine, a universal methyl group donor, which participates in the methylation-dependent biosynthesis of DNA, RNA and protein; (3) it is phosphorylated to synthesize phosphatidylcholine, a major constituent of cell and mitochondrial membranes, which is involved in the mitochondrial bioenergetics regulating lipid and glucose metabolism [1,2,3] and participates in the packaging and exporting of triglyceride (TG) in very low density lipoprotein (VLDL), as well as the solubilizing of bile salts for secretion [2,3,4].

Choline deficiency (CD) contributes to various disorders in animals and humans, with liver as its main target [5]. Humans deprived of choline have been observed to develop fatty liver, liver cell death or skeletal muscle damage [2,6,7], which were further evidenced by another clinical study showing that patients fed with total parenteral nutrition (TPN) solutions low in choline resulted in TPN-associated liver disease [2,8]. Likewise, mouse models with knock-out genes essential for utilizing choline as a methyl donor or synthesizing the choline moiety endogenously also developed liver steatosis [2,9,10].

Growing evidence has suggested specific effects of choline on mitochondrial metabolism. Low choline results in the altered composition of mitochondrial membranes, reduced mitochondrial membrane potential (Δψm) [2,11], decreased ATP production [2,12] and perturbation in fatty acid β-oxidation in rats fed a choline-deficient diet [2,13]. This mitochondrial dysfunction has also been identified in the process of the increment of reactive oxygen species (ROS), the loss of Δψm, cellular apoptosis and hepatocarcinogenesis induced by choline deficiency in SV40-immortalized rat hepatocytes (CWSV-1) [14,15]. Additionally, choline has been evidenced to modify epigenetic marks on genes potently [2,16,17]. Several genes that play a vital role in the pathophysiology of metabolic diseases have to be epigenetically regulated, such as peroxisomal proliferator-activated receptor alpha (PPARα) and PPAR gamma [2,18]. However, the specific mechanisms linking choline, DNA methylation and metabolic diseases, such as non-alcoholic fatty liver disease (NAFLD), remain to be clearly defined.

Excessive energy substrates available to the hepatocytes can potentially cause cellular steatosis with the increasing generation of free fatty acids (FFA) and ROS, which, in turn, will lead to mitochondrial dysfunction inextricably linked with oxidative stress [19,20]. This is central to the development of dietary-induced NAFLD or TPN-associated liver disease under unbalanced nutrients perturbation and can potentially progress to steatohepatitis, fibrosis and cirrhosis in liver [20,21].

Hepatic steatosis is related to the abnormal expression of genes involved in lipid metabolism. It has become clear that the upregulation of gene expression for *de novo* lipogenesis, such as FAS and acetyl-CoA carboxylase, and the downregulation of gene expression for fatty acid oxidation, such as PPARα, carnitine palmitoyl transferase-I (CPT-I) and uncoupling proteins 2 (UCP2), are associated with the onset of hepatic TG accumulation [22]. PPARα can regulate the transcription of a suite of its target genes encoding enzymes in hepatic lipid metabolism, including CPT-I, which are also involved in fatty acid oxidation in liver. Furthermore, DNA methylation in the CpG islands has also been proven to contribute to the regulation of gene expression involved in hepatic lipid metabolism. A recent study has demonstrated that betaine supplement altered DNA methylation modifications on PPARα, as well as the expression of its target genes (CPT-I, UCP2, ACOX, CYP2E) in *ApoE*^−/−^ mice [23].

However, the effects of choline on hepatic mitochondrial impairment, oxidative stress and DNA methylation levels of genes involved in lipid metabolism in human steatosis has not been established yet. To address these questions, we used human C3A cells, a subclone of the hepatoma-derived HepG2 cell line treated with lactate, pyruvate and octanoate to induce TG accumulation and oxidative stress, allowing an exclusive and more efficient mitochondrial β-oxidation, which has been previously adopted as a model for hepatic cellular steatosis [20]. This study was further performed to investigate the effects of choline intervention on TG accumulation, Δψm, the methylation level of the PPARα DNA promoter and the expression of genes involved in hepatic lipid metabolism (FAS, PPARα, CPT-I) by using an *in vitro* hepatocellular steatosis model.

## 2. Experimental Section

### 2.1. Cell Cultures

C3A cells (American Type Culture Collection, Manassas, VA, USA) were grown in minimal essential medium Eagle (MEME) (Sigma-Aldrich, St. Louis, MO, USA) containing 10% bovine serum albumin (BSA) (Sigma-Aldrich, St. Louis, MO, USA), 100 IU/mL penicillin and 100 mg/mL streptomycin (Sigma-Aldrich, St. Louis, MO, USA) at 37 °C in a 5% CO_2_ humidified atmosphere until 70% confluency. LOP-defined medium refers to the above medium supplemented with combinations of lactate (10 mM, L), octanoate (2 mM, O) and pyruvate (1 mM, P) (Sigma-Aldrich, St. Louis, MO, USA). LOP-induced hepatocellular steatosis was established according to Lockman’s method [20]. The cells were then divided into 8 groups after confluency: control (untreated), LOP, choline (5 μM), choline (35 μM), choline (70 μM), LOP + choline (5 μM), LOP + choline (35 μM) and LOP + choline (70 μM) (Sigma-Aldrich, St. Louis, MO, USA). Cells in these groups were cultured for 72 h (unless specified) prior to experimentation.

### 2.2. Cell Viability

Cells were harvested at 24, 48 and 72 h after treatment and then seeded in 96-well plates at 2 × 10^3^ cells/well in 100 μL medium in triplicate. Cell viability was estimated using the cell counting kit-8 (Sigma-Aldrich, St. Louis, MO, USA) according to the manufacturer’s instructions. Cells in each well were supplemented with 10% cell counting kit-8 of 100 μL and incubated for 2 h. Wells with medium alone (no cells) were used as blank. The value of absorbance was obtained at 450 nm (A_450_) and then measured by a microplate reader (Synergy HT, Winooski, VT, USA). The percentage of cell viability was expressed as (A_450_ in treatment − A_450_ in blank)/(A_450_ in control − A_450_ in blank). All experiments were conducted in triplicate and repeated at least three times.

### 2.3. Cellular TG Quantification

Cells in each group were harvested after 72 h of culture. Cellular TG quantification was measured by a TG Quantification Kit (K622-100, BioVision, Mountain View, CA, USA). Briefly, 50 μL TG reaction mix (assay buffer, 46 μL; probe, 2 μL; enzyme mix, 2 μL) was added to each well containing the TG standard, test samples and controls. Then, the reaction solution was mixed well and incubated at room temperature for 60 min in the dark. After that, the fluorescence intensities at Ex. 535 nm and Em. 590 nm were measured by a fluorescence spectrophotometer (Hitachi-F-7000, Tokyo, Japan). Additionally, cellular protein was quantified using BCA protein assay reagents (Thermo Fisher Scientific Inc. Rockford, IL, USA) following the manufacturer’s instructions. The TG concentration was expressed as Ts/Sv where Ts refers to the TG amount from the standard curve and Sv is the sample volume (before dilution) added in sample wells. The results of cellular TG quantification was presented as μg/mg of total protein.

### 2.4. ROS Levels in C3A Cells

The oxidation-sensitive fluorescent probe, dichlorodihydrofluorescein diacetate (DCFH-DA) (Sigma-Aldrich, St. Louis, MO, USA), was used to evaluate the production of intracellular ROS. After being cultured with or without treatment for 72 h, the cells were incubated in 1 mL of reaction buffer containing 10 μM DCFH-DA in a dark atmosphere of 5% CO_2_ at 37 °C for 30 min. The cells were then rinsed with the same buffer, and fluorescence intensity was detected at excitation wavelength 485 nm and emission wavelength 530 nm using a fluorescence spectrophotometer (Hitachi-F-7000, Tokyo, Japan). After that, the cell samples were pelleted, and cellular protein was then quantified using the BCA protein assay reagents (Thermo Fisher Scientific Inc., Rockford, IL, USA) following the manufacturer’s instructions. The results of ROS levels were normalized per protein content, and the ROS levels under choline and LOP treatment were compared with that in the control respectively, with, the data expressed as the percentage of the fluorescence intensity of the control.

### 2.5. Mitochondrial Membrane Potential (ΔΨm) in C3A Cells

Measurement of Δψm was performed using a fluorescent cationic dye, Rhodaminel23 (Rh123, Sigma-Aldrich, St. Louis, MO, USA), which was described previously with minor modifications [24,25]. In brief, C3A cells were incubated with Rh123 (1 μM) for 10 min at 37 °C in an atmosphere of 5% CO_2_. The cells were then rinsed with the reaction buffer, and then, the fluorescence intensity was detected at Ex. 490 nm and Em. 520 nm at 37 °C using a fluorescence spectrophotometer (Hitachi-F-7000, Tokyo, Japan), after which, the cell samples were pelleted to normalize for protein content among samples by the BCA protein assay reagents (Thermo Fisher Scientific Inc., Rockford, IL, USA). The results of Δψm were normalized per protein content, and the percentage of fluorescence intensity was calculated as the ratio of Δψm levels in choline and/or LOP treatments to that in the control, respectively.

### 2.6. Bisulfite Conversion of DNA

Genomic DNA (gDNA) was extracted from cells in each group with the QiaAmp Genomic DNA Kit (Catalog No. 51304, Qiagen, Valencia, CA, USA) as described by the manufacturer. The isolated gDNA was then quantified by a spectrophotometer (NanoDrop 2000, Wilmington, DE, USA) with the gDNA confirmed by 2% agarose gel electrophoresis. An EZ DNA Methylation-Gold kit (catalog No. D5005, Zymo Research, Irvine, CA, USA) was used to prepare bisulfite-modified gDNA according to the manufacturer’s instructions. Briefly, 0.5 μg gDNA (20 μL volume) was added to sample tubes to conduct the bisulfite reaction at a final volume of 150 μL with 130 μL of conversion reagent. The sample tubes were then put in a T100™ Thermal Cycler (Bio-Rad, Hercules, CA, USA) following steps of 10 min at 98 °C and 150 min at 64 °C. Following the incubation, the M-binding buffer (600 μL) was mixed with the converted samples in a Zymo-Spin ICTM Column (Irvine, CA, USA) and then centrifuged at full speed for 30 s, and the flow-through was poured out. One hundred microliters of M-wash buffer were added to rinse the column, and then, the flow-through was discarded after centrifugation at full speed, which was followed by adding 200 μL of M-desulfonation buffer to the column and maintaining it for 20 min at 25 °C. The flow-through was poured out after being centrifuged at full speed for 30 s. Two hundred microliters of M-wash buffer were added to the column and then centrifuged at full speed for 30 s. The above step was repeated for the second time. The bisulfite-converted gDNA samples were then eluted with 10 μL of M-elution buffer, respectively, and kept at −20 °C until required.

### 2.7. Real-Time Quantitative Methylation-Specific PCR

The methylation level of the selected CpG dinucleotides in the PPARα promoter was measured by the methylation-specific PCR (MSP) method using the bisulfite-converted gDNA as a template. The DNA sequence for the human PPARα gene was retrieved from the NCBI website with the accession number in GenBank: NC_000022.10. Two pairs of specific primers were designed to amplify either methylated DNA (M primers) or unmethylated DNA (U primers) via MethPrimer on-line and the sequences are listed in Table 1. Real-time quantitative PCR was carried out using the SYBR^®^ Premix Ex Taq TM II Kit (Takara, Shiga, Japan) in a total volume of 20 μL with 2 μL of converted gDNA, 10 μL 2 × SYBR^®^ Premix Ex Taq TMII, 0.4 μL of 50 ×SYBR Green Master, 0.2 μL forward primer (10 μM), 0.2 μL reverse primer (10 μM) and 7.2 μL water. The amplification was initially conducted with denaturation at 94 °C for 3 min, followed by 40 cycles at 94 °C for 30 s, 55 °C for 30 s and a final extension at 72 °C for 5 min. Each sample was tested in triplicate. A melt curve was then generated to verify the specificity of the PCR product after amplification. A calibration curve was established through serial dilutions of fully methylated and unmethylated, amplified PCR product. Data were collected and analyses by a 7500 Fast Real-Time PCR System (Applied Biosystems, Foster City, CA, USA) and its software V2.0.6. The methylation percentage for each sample was estimated as the mean value of mC/(mC + C) for all examined CpGs in the gene. The fully unmethylated and methylated positive controls were performed by using unmethylated and fully methylated NIH 3 T3 mouse genomic DNA (New England Biolabs, Ipswich, MA, USA), respectively. The negative control was also included without the addition of the PCR products.

**Table 1:** Sequences of amplification primers for detecting gene promoter methylation and mRNA expression.

Gene Symbol	Primer Sequence (5′–3′)	Anneal Temperature	Amplicon Size
PPARα M	F:GGTTTTAGGGATAAGGTTTTTTTC	57	122
	R:ACCCGACTCTACTACTCTATACGAT		
PPARα U	F:GGTTTTAGGGATAAGGTTTTTTTTG	55	122
	R:ACCCAACTCTACTACTCTATACAAT		
FAS	F:TATGCTTCTTCGTGCAGCAGTT	55	98
	R:GCTGCCACACGCTCCTCTAG		
PPARα	F:GCAGAAACCCAGAACTCAGC	58	141
	R:ATGGCCCAGTGTAAGAAACG		
CPT-I	F:GATTTTGCTGTCGGTCTTGG	56	123
	R:CTCTTGCTGCCTGAATGTGA		
18S	F:CGGCTACCACATCCAAGGAA	64	186
	R:GCTGGAATTACCGCGGCT		

M: methylated; U: unmethylated; F: Forward primer; R: Reversed primer.

### 2.8. Quantitative Analysis of Gene Expression

Real-time PCR was conducted for evaluating mRNA expression of PPARα, FAS and CPT-I, respectively. Total RNA was isolated from the cells in each group using the RNeasy Mini Kit (Catalog No. 74104, QIAGEN, Valencia, CA, USA) following the manufacturer’s instructions, with the concentration and quality of total RNA measured by the spectrophotometer (NanoDrop. 2000, Wilmington, DE, USA) at OD_260_ nm and its integrity assessed on agarose gels. An equal concentration of RNA (1 μg) was taken from all of the samples, and quantitative analysis of gene expression was carried out in a total volume of 25 μL using the One Step SYBR PrimeScript RT-PCR Kit II (Takara, Shiga, Japan) following the manufacturer’s instructions. The amplification was initially carried out with denaturation at 94 °C for 3 min, followed by 40 cycles at 94 °C for 30 s, 55 °C for 30 s and a final extension at 72 °C for 5 min.

Oligonucleotide primers for each target gene are summarized in Table 1. 18S was adopted as the endogenous reference to normalize the transcript levels. Samples were assayed with a fast, real-time PCR system (Applied Biosystems 7500, Foster City, CA, USA).

### 2.9. FAS Activity in C3A Cells

The enzymatic activity of FAS was measured using the FAS commercial kit (Jiancheng Bioengineering Institute, Nanjin, China) according to the method, as described previously [26]. Briefly, particle-free supernatants were obtained after the cells were sonicated at 4 °C and centrifuged at 12,000× *g* for 30 min. A total volume of 500 μL of the assay mixture containing 125 μL particle-free supernatant, 25 mM KH_2_PO_4_–K_2_HPO_4_ buffer, 0.25 mM EDTA, 0.25 mM dithiothreitol, 30 mM acetyl-CoA and 350 mM NADPH (pH 7.0) were monitored at 340 nm for 1 min at 37 °C to measure background NADPH oxidation. Immediately prior to measurements, the assay mixture was added with 100 mM malonyl-CoA for an additional 3 min of incubation, and the change in absorbance at 340 nm was recorded with a spectrophotometer (Hitachi-F-7000, Tokyo, Japan). FAS activity was expressed as nmol oxidized NADPH/min/mg protein.

### 2.10. CPT-I Activity in C3A Cells

The enzymatic activity of CPT-I was assayed using the CPT-I commercial kit (Jiancheng Bioengineering Institute, Nanjin, China) according to the instructions. The amount of CoA-SH released from palmitoyl CoA was actually quantified by measuring an absorbance at 412 nm to demonstrate CPT-I activity. Briefly, particle-free supernatant was prepared from the cells and firstly incubated with 5,5′-dithio-bis-(2-nitrobenzoic acid) at room temperature for 30 min. The assay mixture containing 100 μM palmitoyl-CoA, 5 mM L-carnitine solution and 1 M Tris (pH 8.0) was then added to start the reaction. The change in absorbance at 412 nm at 37 °C was immediately recorded with a spectrophotometer (Hitachi-F-7000, Tokyo, Japan). Protein concentrations of cleared lysates were measured using the BCA protein assay. CPT-I activity was expressed as nmol CoA-SH released/min/mg protein.

### 2.11. GSH-Px Activity in C3A Cells

Enzymatic activity of GSH-Px was evaluated using the GSH-Px commercial kit (Jiancheng Bioengineering Institute, Nanjin, China) according to the instructions. Briefly, the cytosolic supernatant was prepared from the cells after centrifugation at 8000× *g* for 20 min at 4 °C and was added to the prewarmed reaction mixture (0.1 M GSH, 10 IU/mL GSH-Rd, 1 M Tris, 5 mM EDTA buffer, 2 mM NADPH and 7 mM tert-butyl hydroperoxide, pH 8.0.). GSH-Px activity was determined by monitoring NADPH oxidation at 340 nm at 37 °C immediately with a spectrophotometer (Hitachi-F-7000, Tokyo, Japan). Protein concentrations of cleared lysates were measured using the BCA protein assay (Thermo Fisher Scientific Inc., Rockford, IL, USA). GSH-Px activity was expressed as nmol of oxidized NADPH /min/mg protein.

### 2.12. Data Analysis

All results were presented as the mean ± standard deviation (SD) and statistical analyzed by using SPSS software version 16.0 (SPSS Inc., Chicago, USA) for Windows. Differences between groups were evaluated by one-way ANOVA followed by Bonferroni’s multiple comparison. A two-way ANOVA was conducted for cell viability to determine the interaction between the specified treatment and the duration of treatment. A two-tailed *p* < 0.05 was considered statistically significant.

## 3. Results

### 3.1. Effects of LOP and Choline on Cell Viability

Cells cultured in LOP-treated medium alone exhibited decreased cell viability compared to that in the control at 48 h and 72 h, respectively (*p* < 0.05). Additionally, choline treatment (5, 35 or 70 μM) alone exerted no effects on cell viabilities compared with that in the control, respectively. In contrast, cell viability was restored when grown in LOP-treated medium containing choline (35 μM or 70 μM) at 48 h and 72 h, respectively (*p* < 0.05), although concomitant treatment of low choline (5 μM) and LOP did not change cell viability relative to that in LOP medium alone. Moreover, there was no significantly difference in cell viability when cells were cultured in LOP medium containing 35 μM choline compared to that in LOP medium containing 70 μM choline (*p* > 0.05, Table 2).

**Table 2:** Effects of choline and LOP on C3A cell viability (%).

Groups	24 h	48 h	72 h
Control	95.9 ± 1.2	97.1 ± 1.9	98.2 ± 2.1
LOP	95.2 ± 1.1	92.8 ± 1.3 *	90.9 ± 1.7 *
CH (5 μM)	95.8 ± 1.2	97.2 ± 2.1	98.3 ± 1.9
LOP + CH (5 μM)	95.4 ± 0.9	93.2 ± 1.4 *	91.1 ± 1.6 *
CH (35 μM)	96.1 ± 1.4	97.5 ± 1.7	98.9 ± 2.5
LOP + CH (35 μM)	95.4 ± 1.5	96.4 ± 1.6	97.3 ± 2.0
CH (70 μM)	96.2 ± 1.6	97.5 ± 1.9	98.9 ± 2.3
LOP + CH (70 μM)	95.4 ± 1.7	96.3 ± 1.8	97.5 ± 1.9

CH, choline; LOP, lactate (10 mM), octanoate (2 mM) and pyruvate (1 mM); values represent the mean ± SD; * *p* < 0.05 *vs.* the control.

### 3.2. Effects of LOP and Choline on TG Accumulation in C3A Cells

**Figure 1:**
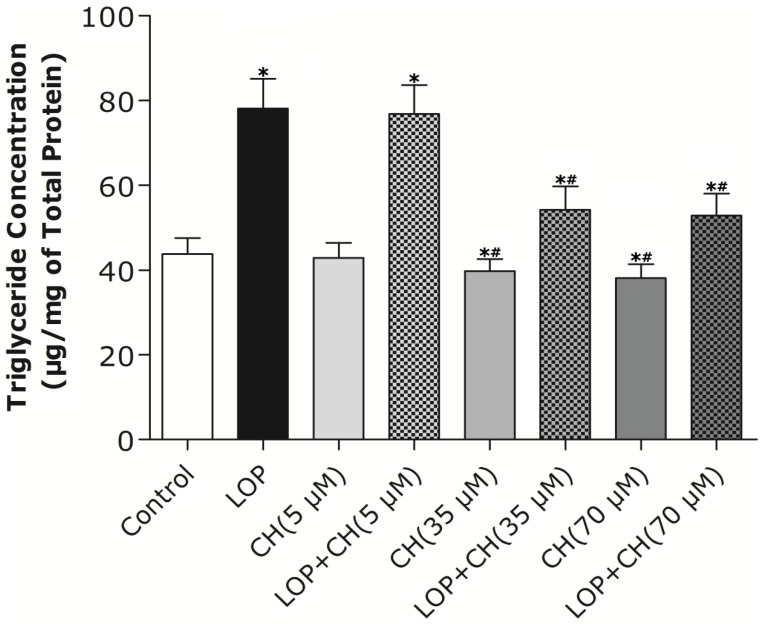
The effects of LOP and choline on TG accumulation. C3A cells were cultured in MEME with or without LOP and choline (5, 35, 70 μM) treatment for 72 h. Hepatic TG content was measured by a TG Quantification kit through a fluorescence spectrophotometer. CH, choline; LOP, lactate (10 mM), octanoate (2 mM) and pyruvate (1 mM). Values represent the mean ± SD of three independent experiments performed in duplicate. * *p* < 0.05 *vs.* control. ^#^ *p* < 0.05 *vs.* LOP.

Treatment with LOP resulted in higher intracellular TG accumulation at 72 h than that in the control, as shown in Figure 1 (*p* < 0.05). The low dose of choline (5 μM) treatment alone exhibited no effect on TG accumulation when compared with that in the control, but both the 35 μM and 70 μM choline treatments alone decreased TG accumulation with no marked differences between these two concentrations. Conversely, the addition of 35 μM or 70 μM choline to LOP attenuated TG accumulation at 72 h, respectively, and similarly, relative to those in the LOP treatment alone (*p* < 0.05), although concomitant treatment of LOP and choline (5 μM) did not reverse the increase of TG accumulation compared with that in the LOP treatment alone (Figure 1).

### 3.3. Effects of LOP and Choline on the ROS Level in C3A Cells

**Figure 2:**
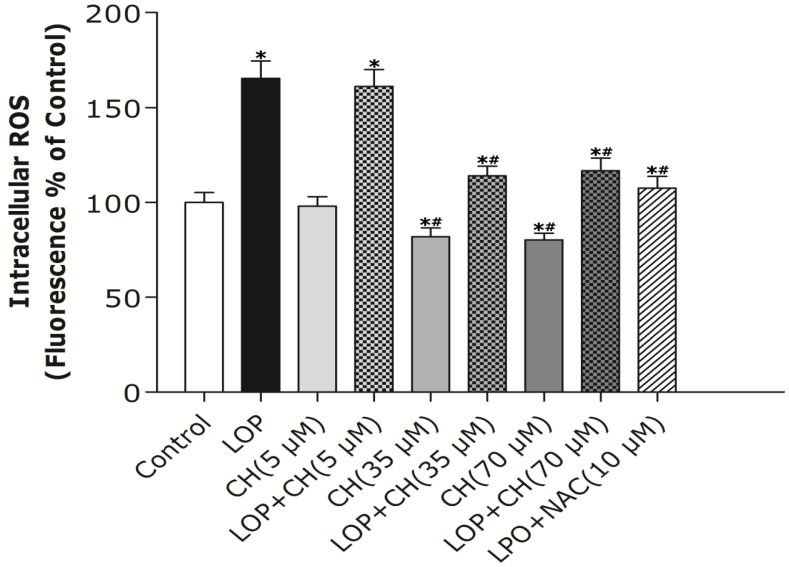
The effects of LOP and choline on intracellular ROS formation. C3A cells were cultured in MEME with or without LOP, choline (5, 35, 70 μM) and NAC (10 mM) treatment for 72 h. The production of intracellular ROS was determined by using dichlorodihydrofluorescein diacetate (DCFH-DA) as a fluorescent probe through a spectrofluorometer. CH, choline; LOP, lactate (10 mM), octanoate (2 mM) and pyruvate (1 mM). Values represent the mean ± SD of three independent experiments performed in duplicate. * *p* < 0.05 *vs.* control. ^#^ *p* < 0.05 *vs.* LOP.

To examine the impact of extra energy substrates on the generation of ROS, LOP was added to MEME medium throughout the 72-h cell culture. ROS formation was increased by about 65% in LOP-treated cells when compared with the control, as shown in Figure 2 (*p* < 0.05). Additionally, the low-choline (5 μM) treatment alone manifested no influence on ROS formation compared to that of the control, but the 35 μM or 70 μM choline treatment alone decreased ROS formation by about 34%, respectively, relative to that in the control (*p* < 0.05).

As it has been established that *N*-acetyl-l-cysteine (NAC), an effective anti-oxidant, can enhance hepatic mitochondrial function and modulate ROS formation [20], we combined NAC (10 mM) with LOP treatment. Concomitant treatment with NAC inhibited the increase of ROS formation in LOP-treated cells in comparison with LOP alone (*p* < 0.05). Similarly, the combination of LOP with choline at a dose of 35 μM or 70 μM reduced the ROS formation by about 30%, respectively, relative to LOP treatment alone (*p* < 0.05), whereas concomitant treatment with LOP and choline (5 μM) did not attenuate the increase of ROS production caused by LOP (Figure 2).

### 3.4. Effects of LOP and Choline on Δψm in C3A Cells

**Figure 3:**
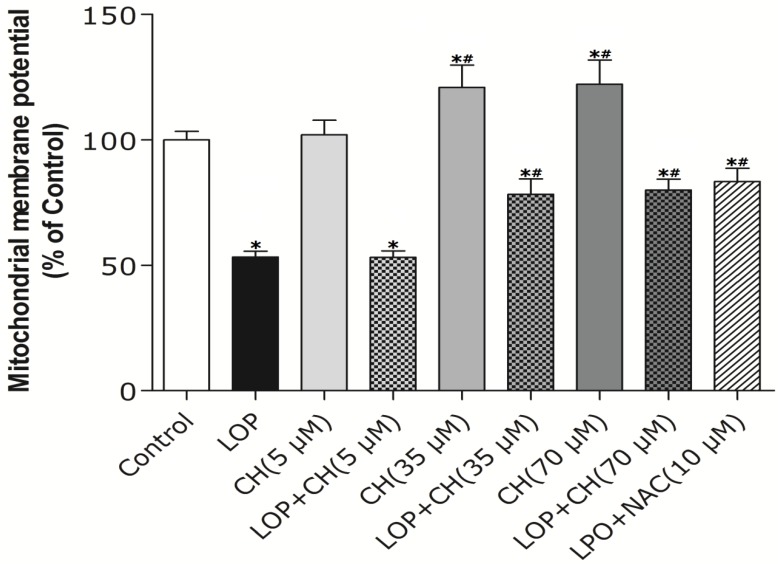
The effects of LOP and choline on Δψm. C3A cells were cultured in MEME with or without LOP, choline (5, 35, 70 μM) and NAC (10 mM) treatment for 72 h. Δψm was measured by fluorescence analysis using Rhodaminel23 as a fluorescent cationic dye through a spectrofluorometer. CH, choline; LOP, lactate (10 mM), octanoate (2 mM) and pyruvate (1 mM). Values represent the mean ± SD of three independent experiments performed in duplicate. * *p* < 0.05 *vs.* control. ^#^ *p* < 0.05 *vs.* LOP.

As Δψm plays a vital role in modulating ROS formation, it was then measured in C3A cells by using Rhodaminel23 fluorescence analysis. As demonstrated in Figure 3, the low-choline (5 μM) treatment alone exhibited a similar effect on Δψm in C3A cells as that in the control, whereas the 35 μM or 70 μM choline treatment alone enhanced Δψm by about 28% relative to that in the control (*p* < 0.05). Additionally, LOP treatment resulted in a significantly lower ΔΨm compared with that in the control (*p* < 0.05). Furthermore, concomitant treatment with antioxidant NAC (10 mM) attenuated the decrease of Δψm in LOP-treated cells (*p* < 0.05). Similarly, Δψm was about 50% higher upon concomitant treatment with LOP and choline at a concentration of 35 μM or 70 μM, respectively, when compared to LOP alone (*p* < 0.05), whereas concomitant treatment with LOP and choline (5 μM) did not abolish the decrement of Δψm induced by LOP (Figure 3).

### 3.5. Effects of LOP and Choline on the mRNA Expression of FAS, PPARα and CPT-I Genes in C3A Cells

**Figure 4:**
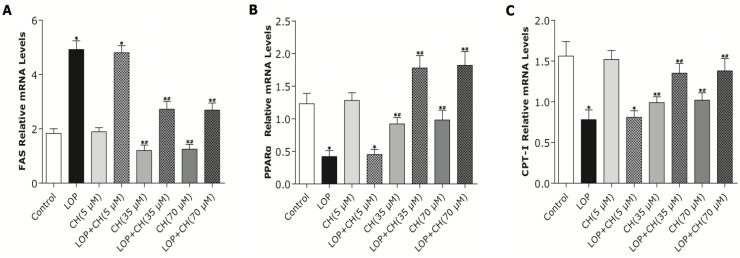
The effects of LOP and choline on mRNA expression of FAS, PPARα and CPT-I genes. C3A cells were cultured in MEME with or without LOP and choline (5, 35, 70 μM) treatment for 72 h. (**A**–**C**) The mRNA expression of FAS, PPARα and CPT-I genes was determined by a fast, real-time PCR system. CH, choline; LOP, lactate (10 mM), octanoate (2 mM) and pyruvate (1 mM). Values represent the mean ± SD of three independent experiments performed in duplicate. * *p* < 0.05 *vs.* control. ^#^ *p* < 0.05 *vs.* LOP.

The low-choline (5 μM) treatment alone exhibited no effect on the expression of FAS, PPARα and CPT-I genes in C3A cells when compared with those in the control, respectively (Figure 4). However, the 35 μM and 70 μM choline treatments exerted similar effects, which were evidenced as the reduced mRNA expression of FAS, PPARα and CPT-I genes in C3A cells by about 33%, 25% and 35%, respectively, compared to those in the control (*p* < 0.05). Hepatic FAS mRNA expression in LOP-treated C3A cells was significantly higher than that of the control (*p* < 0.05), while concomitant treatment of LOP and choline at 35 μM or 70 μM decreased its expression levels by about 45%, respectively, when compared with LOP alone (*p* < 0.01). In contrast, concomitant treatment of LOP and choline (5 μM) did not reduce the expression level of FAS mRNA relative to LOP alone (Figure 4). Additionally, exposure to LOP treatment reduced PPARα and CPT-I mRNA expression when compared to those in the control (*p* < 0.05), which were upregulated by about 333% and 77%, respectively, upon concomitant treatment with LOP and choline at 35 μM or 70 μM in C3A cells, relative to LOP alone (*p* < 0.01), whereas concomitant treatment of LOP and choline (5 μM) did not alter PPARα and CPT-I mRNA expression, compared to LOP alone (Figure 4).

### 3.6. Effects of LOP and Choline on the Methylation Level of the PPARα Gene Promoter in C3A Cells

**Figure 5:**
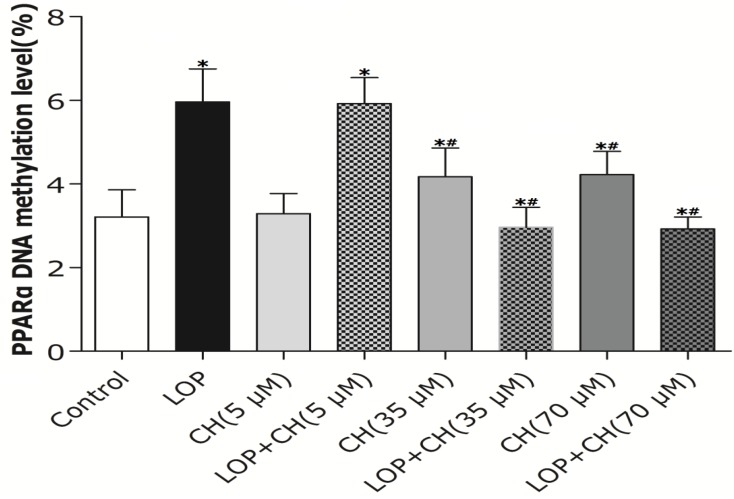
The effects of LOP and choline on the methylation level of the PPARα gene promoter. C3A cells were cultured in MEME with or without LOP and choline (5, 35, 70 μM) treatment for 72 h. The methylation level of PPARα gene promoter was measured by real-time quantitative MSP. CH, choline; LOP, lactate (10 mM), octanoate (2 mM) and pyruvate (1 mM). Values represent the mean ± SD of three independent experiments performed in duplicate. * *p* < 0.05 *vs.* control. ^#^ *p* < 0.05 *vs.* LOP.

As PPARα can regulate the transcription of its target genes encoding enzymes, such as CPT-I, involved in hepatic mitochondrial fatty acid oxidation and choline plays an important role in the DNA methylation, a real-time quantitative MSP method was adopted to measure the methylation levels of the PPARα promoter region quantitatively. The low-choline treatment (5 μM) alone exhibited no effect on the average methylation levels in the PPARα gene promoter region, whereas the 35 μM and 70 μM choline treatment alone enhanced it by 30% of that in the controlled cells, respectively (*p* < 0.05, Figure 5). Additionally, the average methylation levels in the PPARα gene promoter region in C3A cells enhanced from 3.21% ± 0.65% in the controlled cells to 5.96% ± 0.79% in LOP-treatment cells (*p* < 0.05). In contrast, the combination of LOP with choline at 35 μM or 70 μM attenuated the average methylation levels by 50%, respectively, compared to LOP treatment alone (*p* < 0.05), whereas concomitant treatment of LOP and choline (5 μM) did not alter the average methylation levels in the PPARα gene promoter region in C3A cells when compared to that in the LOP treatment alone. These declined methylation levels of the PPARα promoter were also accompanied by enhanced PPARα gene expression upon treatment with choline (Figure 4 and Figure 5).

### 3.7. Effects of LOP and Choline on FAS, CPT-I and GSH-Px Activities in C3A Cells

**Figure 6:**
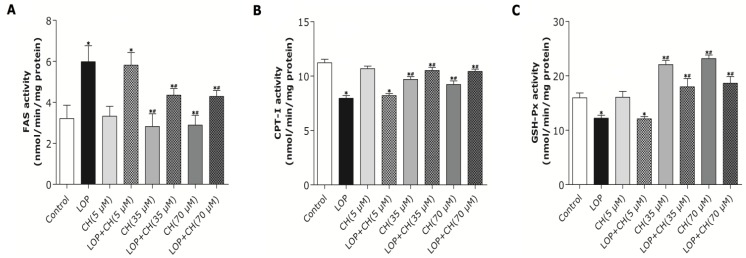
The effects of LOP and choline on FAS, CPT-I and GSH-Px activity. C3A cells were cultured in MEME with or without LOP and choline (5, 35, 70 μM) treatment for 72 h. (**A**–**C**) The enzyme activity of FAS, CPT-I and GSH-Px was determined by the spectrophotometric assay, respectively. CH, choline; LOP, lactate (10 mM), octanoate (2 mM) and pyruvate (1 mM). Values represent the mean ± SD of three independent experiments performed in duplicate. * *p* < 0.05 *vs.* Control. ^#^ *p* < 0.05 *vs.* LOP.

Treatment with choline at 35 μM or 70 μM alone attenuated FAS and CPT-I activity by 10% and 35% and enhanced GSH-Px activity by 46%, approximately (*p* < 0.05, Figure 6), with no significant difference between these two concentrations, whereas the low-choline (5 μM) treatment did not alter these enzymes activities when compared to the control. LOP treatment enhanced FAS activity by 86%, but reduced CPT-I and GSH-Px activity by 29% and 23%, approximately, after 72 h of culture when compared with the control (*p* < 0.05, Figure 6). Contrarily, concomitant treatment with LOP + choline at 35 μM or 70 μM counteracted the LOP effects, with FAS activity abolished by 28% and CPT-I and GSH-Px activities elevated by approximately 29% and 52%, equally and respectively, when compared to those in the LOP treatment alone (*p* < 0.05). However, restoration of the above enzyme activities were not observed in C3A cells under concomitant treatment with LOP + choline (5 μM) relative to LOP treatment alone (Figure 6).

## 4. Discussion

The present study was designed to determine the effects of choline on modulating hepatic TG accumulation, DNA methylation of PPARα, mRNA expression of the critical genes and their enzyme activities involved in hepatic lipid metabolism, ROS levels, Δψm and GSH-Px activity through *in vitro* models of cellular steatosis using a combination of excessive energy substrates (lactate, pyruvate and octanoate, LOP). In the current study, we adopted the LOP-induced cellular steatosis models in C3A cells, because it has been proven to recapitulate the pivotal features relevant to steatohepatitis, such as enhanced TG content and ketogenesis, altered glucose metabolism, increased oxidative stress and impaired mitochondrial function [20], which are associated with human nonalcoholic fatty liver disease with excess nutrients in diet [20,27]. Additionally, choline chloride (5, 35 and 70 μM) was adopted to culture C3A cells with or without LOP concomitant treatment to investigate the biological effects by choline itself and its role upon LOP perturbation.

The concentration of choline in the original MEME is about 7.16 μM according to the MEME formulation (Sigma-Aldrich, St. Louis, MO, USA). There was no marked alteration in cell viability when cells were incubated with 5 μM, 35 μM or 70 μM choline alone for 72 h, suggesting that choline alone does not alter the cells survivability in MEME. However, the cell viability was significantly different between the untreated cells and LOP treated or LOP + choline (5 μM) treated cells, but a high dose of choline (35 or 70 μM) reversed the deleterious effect of LOP on C3A cells viability, indicating that hepatic cells may need more choline in the MEME to protect them from continuous detrimental influence under the surge of energy substrates.

Choline, which is used to synthesize phosphatidylcholine essential for the packaging, exporting and secreting of TG in VLDL, plays an important role in mediating hepatic steatosis [20,27,28,29]. Previous studies have demonstrated that mouse models with the deletion of genes in choline metabolism develop fatty liver [2,9]. Additionally, humans fed low-choline diets have also been evidenced to develop fatty liver [7,30]. Even the daily recommended intakes of choline for the common population may not meet the requirement for patients with fatty liver disease to prevent the development of liver steatosis [2]. However, the specific mechanisms of choline intervention on the formation of hepatic steatosis under excessive energy substrate perturbation still remain unclear. Therefore, we have cultured C3A cells with LOP for 72 h to induce cellular steatosis and mitochondrial dysfunction.

Our results (Figure 2) characterized the ability of 35 or 70 μM choline, but not 5 μM choline, to decrease TG content in C3A cells by itself or with concomitant treatment of LOP. We further found out that choline diminished the increased TG accumulation by about 32% and attenuated FAS mRNA expression and its activity in LOP-treated C3A cells, thus suggesting that under excessive energy substrate perturbation, choline is capable of inhibiting fatty acid synthesis, which is essential for lipogenesis, besides its abilities of accelerating the export and secretion of TG [2].

Mitochondrial dysfunction is evidenced as a central mechanism in the pathogenesis of NAFLD, featuring ROS formation and decreased Δψm [2,19,20]. Choline deficiency has also been characterized as leading to ROS generation, which was followed by the loss of ΔΨm and decreased ATP production in CWSV-1 cells [14,31,32]. Therefore, our study intends to find out whether more choline is needed and how choline intervention can prevent mitochondria from impairment in C3A cells exposed to excessive energy substrates. Our results demonstrated that LOP treatment obviously increased ROS formation and caused mitochondria impairment in C3A cells, which is consistent with the result of Lockman *et al.* [20]. Additionally, our results manifested that NAC, a potent antioxidant, prevented the C3A cells from the increase of ROS and the decrease of Δψm in LOP-cultured cells at 72 h, respectively (Figure 2 and Figure 3), suggesting that oxidative stress is the cause leading to mitochondrial impairment in excessive energy substrate perturbation. We also found out that 35 μM or 70 μM choline alone has equal protective effects on the mitochondria in C3A cells with enhanced Δψm. One of the other physiological functions that choline exerts is to synthesize phosphatidylcholine, a major constituent of the mitochondrial membrane, which relates to the mitochondrial bioenergetic functions [1,2,3]. Therefore, the increased Δψm induced by choline treatment alone on C3A cells may be due to the possibility that choline maintains mitochondrial membrane integrity, prevents it from impairment caused by ROS and promotes its functions involved in bioenergetics, as well as electron transfer.

Akin to the findings in NAC coculture, ROS formation and altered Δψm in LOP-treated cells were also markedly mitigated by 35 μM or 70 μM choline intervention, respectively, indicating that choline protects mitochondria from impairment through alleviating oxidative stress. It has been reported that choline deficiency leads to impaired mitochondrial respiratory chain function, particularly complexes I and III dysfunction in CWSV-1 cells, due to an elevation in ROS production [14,15,33]. It is also known that abnormal ROS formation caused by excessive energy substrates results in lipid peroxidation, which changes mitochondrial permeability, followed by the loss of Δψm and the reduction of ATP [2,3,4,5,31]. This alteration promotes further ROS formation and eventually further aggravates impairment on mitochondria with initiating cellular apoptosis that progresses from hepatic steatosis to steatohepatitis, fibrosis, cirrhosis and liver carcinoma [2,3,34]. Previous studies have also demonstrated that choline deficiency changes the composition of mitochondrial membranes which makes it become leaky, due to the overproduction of free radicals, leading to mitochondrial dysfunction in hepatocytes [2,3,5,14,15,34]. Therefore, it is plausible that more ROS formation arising from LOP treatment is attributed to lipid peroxidation, which resulted from the fact that choline in the original MEME may not be enough to maintain the integrity of mitochondrial membranes under excessive energy substrate perturbation. Concomitant treatment with extra choline (35 μM or 70 μM) in LOP-treated C3A cells may protect the mitochondrial membrane from damage caused by LOP through decreasing ROS generation, thus increasing ΔΨm and preventing mitochondrial impairment.

It has been established that gene expression involved in lipid metabolism is affected by hepatic steatosis [22,23]. PPARα is a well-known modulator involved in lipid and carbohydrate metabolism, with its abundant expression in the liver [35,36]. Hepatic PPARα expression has been documented to be downregulated in NAFLD patients [37]. PPARα is able to promote lipid catabolism through upregulation of its target genes, such as CPT-I and CYP2E1, which are involved in fatty acid oxidation in mitochondria and extramitochondria [35,36,37]. In the present study, both the 35 and 70 μM choline treatments alone decreased PPARα gene expression, but significantly upregulated PPARα gene expression in LOP-treated C3A cells. The expression of CPT-I, one of the targets of PPARα and a key rate-limiting enzyme in mitochondrial oxidation, was just paralleled by the enhanced PPARα expression in response to choline intervention.

However, how PPARα regulates its downstream target genes precisely still remains unclear. Recently, Wang’s results have shown that betaine, the downstream metabolite in choline metabolism, is able to upregulate mRNA expression of PPARα and its target genes (CPT-I, CYP2E1) through diminishing hepatic PPARα promoter methylation in *ApoE*^−/−^ mice [22]. It is well established that when the promoter of a gene is heavily methylated, its expression is normally silenced or reduced [22,32]. Moreover, there could be global DNA hypomethylation in cells, but hypermethylation of a particular gene’s promoter also exists concurrently [35,36]. Choline serves as a potent modifier of epigenetic marks in the body for the methylation of DNA, RNA, histone and proteins [2,16,17]. Thus, we further investigated whether the alteration of PPARα promoter methylation and corresponding gene expression can be influenced by choline intervention in LOP-treated C3A cells. Our results showed that both 35 and 70 μM choline alone and LOP treatment enhanced the methylation level of the PPARα gene promoter in C3A cells, respectively, compared to that of untreated cells. In contrast, concomitant treatment of choline (35 or 70 μM) and LOP reversed it by about 52% when compared with that of the LOP treatment alone (Figure 5), which is similar to Wang’s results [22].

However, what is the mechanism by which choline (35 or 70 μM), as a methyl donor, decreased the methylation level of the PPARα gene promoter in C3A cells under LOP perturbation? It has been evidenced that the DNA methylation level of the PPARα promoter was positively associated with the concentration of hepatic betaine in choline metabolism [22]. Choline is catalyzed into betaine by choline dehydrogenase (CHDH) and betaine aldehyde dehydrogenase (BADH), sequentially, in liver, and hepatic CHDH is competitively suppressed by both betaine aldehyde and betaine [22]. Once betaine is generated, it serves as a methyl donor for remethylating homocysteine into methionine by betaine-homocysteine methyltransferase (BHMT). Additionally, it has been established that betaine-elicited inhibition of CHDH and activation of BHMT can collectively enhance catabolism, but decrease the synthesis of betaine in the liver in *ApoE*^−/−^ mice with hepatic steatosis [22,38]. Therefore, hepatic betaine depletion contributes to the hypomethylation of the PPARα promoter [22]. Apart from being a methyl donor, choline also participates in the assembly of mature VLDL as a basic component of the synthesis of phosphatidylethanolamine (PE) and phosphatidylcholine (PC) [22,39]. It has been proven that more choline is essential to enhance PC synthesis and the PC:PE ratio in VLDL in mice with hepatic steatosis, hence promoting VLDL secretion and lipid transport, as well as solubilizing bile salts for secretion [22,39,40]. Therefore, the possible explanation is that choline intervention under LOP perturbation in C3A cells may reduce the concentration of the related metabolites, such as betaine, and more choline may be stored for other physiological functions, like PE and PC synthesis for VLDL formation, which accelerates lipid transport and removal. Thus, not enough substrates may be supplied for DNA methylated modification, which may lead to selective reduction of the methylation level of certain genes, such as PPARα, in lipid metabolism [22]. We further found out that the mRNA expression of PPARα and its target gene, CPT-I, just mirrored the alteration of the PPARα promoter methylation in response to concomitant treatments of LOP and choline (35 or 70 μM), which indicates that choline may accelerate the catabolism of fatty acid by attenuating PPARα promoter methylation, as well as upregulating PPARα and CPT-I mRNA expression under excessive energy substrate perturbation. The comprehensive effects that choline intervention significantly augmented CPT1 mRNA expression and activity, as well as reduced the expression and activity of FAS under LOP perturbation, may impair fatty acid synthesis. Thus, less substrates are supplied for lipid peroxidation, and the elevated mitochondrial oxidation is sufficiently powerful to prevent TG accumulation and oxidative stress.

It has been well documented that over-oxidation involved in hepatic fatty acid metabolism definitely leads to oxidative stress with excessive ROS formation [22,41]. In order to control the detrimental potential of ROS, antioxidant enzymes are available to catalyze free radical-quenching reactions [42]. GSH-Px, a potent hepatic antioxidant enzyme, has been evidenced to scavenge ROS and to participate in the decreased rate of lipid peroxidation under obesity-induced oxidative stress [42]. In the present study, choline (35 or 70 μM) treatment alone increased GSH-Px activity in C3A cells with no effect of 5 μM choline on it. However, decreased GSH-Px activity was observed in LOP-treated C3A cells, but the simultaneous addition of choline (35 or 70 μM) in the LOP treatment strikingly counteracted the decline in the GSH-Px activity (Figure 6), suggesting that choline possess an antioxidant-status improving ability in C3A cells under excessive energy substrate perturbation.

## 5. Conclusions

In conclusion, choline itself exerted protective effects on mitochondrial function, decreasing TG content, as well as enhancing antioxidant capacity in C3A cells. Additionally, LOP treatment, as an excessive energy substrate, had the ability to cause hepatocellular TG accumulation and oxidative stress in C3A cells. Choline also exerted the biological effects of relieving hepatocellular TG deposits, promoting lipid catabolism and ROS scavenging under LOP perturbation, which was partially achieved via reversing the methylation status of the PPARα gene promoter, upregulating PPARα and CPT-I gene expression, decreasing FAS gene expression and its activity, as well as enhancing CPT-I and GSH-Px activity. However, the specific mechanisms linking choline, epigenetics and energy metabolism warrant further investigation. These findings provided a novel insight into the lipotropic role of choline as a vital nutrient of methyl-donation in the intervention of chronic metabolic diseases.
